# Impact of Machine Perfusion on Biliary Complications after Liver Transplantation

**DOI:** 10.3390/ijms19113567

**Published:** 2018-11-12

**Authors:** Andrea Schlegel, Philipp Dutkowski

**Affiliations:** 1 Department of Surgery & Transplantation, University Hospital Zurich, 8091 Zurich, Switzerland; philipp.dutkowski@usz.ch; 2 The Liver Unit, Queen Elizabeth University Hospital Birmingham, Birmingham B15 2TH, UK; 3 NIHR Liver Biomedical Research Unit, University Hospitals Birmingham, Birmingham B15 2TH, UK

**Keywords:** hypothermic oxygenated perfusion (HOPE), cholangiocyte injury, mitochondria

## Abstract

We describe in this review the different types of injuries caused to the biliary tree after liver transplantation. Furthermore, we explain underlying mechanisms and why oxygenated perfusion concepts could not only protect livers, but also repair high-risk grafts to prevent severe biliary complications and graft loss. Accordingly, we summarize experimental studies and clinical applications of machine liver perfusion with a focus on biliary complications after liver transplantation. Key points: (1) Acute inflammation with subsequent chronic ongoing liver inflammation and injury are the main triggers for cholangiocyte injury and biliary tree transformation, including non-anastomotic strictures; (2) Hypothermic oxygenated perfusion (HOPE) protects livers from initial oxidative injury at normothermic reperfusion after liver transplantation. This is a unique feature of a cold oxygenation approach, which is effective also end-ischemically, e.g., after cold storage, due to mitochondrial repair mechanisms. In contrast, normothermic oxygenated perfusion concepts protect by reducing cold ischemia, and are therefore most beneficial when applied instead of cold storage; (3) Due to less downstream activation of cholangiocytes, hypothermic oxygenated perfusion also significantly reduces the development of biliary strictures after liver transplantation.

## 1. Introduction

The ultimate goal and task for new preservation strategies is to treat and repair high-risk organs, which were previously deemed not transplantable. With a steadily increasing amount of data, the transplant community learns that such approaches may enable us to prevent severe reperfusion injury and subsequent complications after liver transplantation. With the improvement of anesthesiological and surgical techniques, the majority of transplant recipients achieve an initial recovery, and the rate of primary-non-function (PNF) is low [1]. However, the major problems in the field remain the biliary complications after liver transplantation, and accordingly there are two main strategies physicians put their focus on [2]. The first is to understand which donor-recipient risk combinations should be avoided and when to say “no” to prevent severe biliary complications [3]. The second is to treat livers before implantation to improve their quality with the use of novel machine perfusion technology [4]. In order to achieve this goal, an understanding of the underlying mechanism of biliary injury in the setting of liver transplantation appears mandatory, which is therefore the first subject of this review. Secondly, we will focus on recent literature including experimental and clinical studies assessing the impact of machine perfusion technology on the biliary tree and on biliary complications after liver transplantation.

## 2. Overview of Biliary Injury and Underlying Mechanism in the Setting of Liver Transplantation

**Figure 1:**
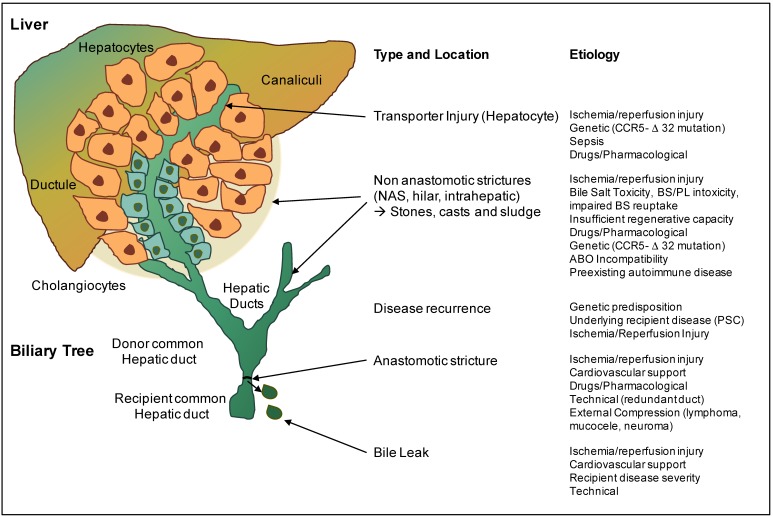
Type and etiology of injury of the biliary tree after liver transplantation. The site and type of biliary complication are highlighted, and potential causes are described throughout the entire biliary tree of a liver graft.

Anastomotic and non-anastomotic biliary strictures (AS and NAS) are the most common biliary complications after liver transplantation, with a frequency of 1–15% and 5–30%, respectively [5,6] (Figure 1). Donation after circulatory death (DCD) livers experience a certain period of warm ischemia in the donor, which has been recognized in combination with cold storage and other risk factors as a main cause of the higher incidence of NAS after DCD liver transplantation [7]. In addition, altered bile acid composition level, triggered by hepatocyte transporter injury and immune-mediated processes play a cumulative role in the pathogenesis of biliary injury and subsequent formation of biliary strictures (Figure 1) [8]. Relatively short periods of ischemia already induce a rapid cellular energy depletion in the very sensitive biliary epithelial cells (cholangiocytes) [9], which subsequently detach from the basement membrane due to a loss of their intercellular connections. In this context, several clinical studies have described a significant loss of epithelial cells in large extrahepatic bile ducts after transplantation of otherwise relatively low-risk livers, donated after brain death (DBD) [10]. Despite the observation that biliary injury is almost universally present (>80%) already before graft implantation, biliary strictures are seen in only a minority of transplant recipients, which has led to the hypothesis that the ability of the bile duct wall and epithelium to regenerate is an important feature of the pathogenesis of biliary strictures. While it is clear that regeneration of the larger bile ducts requires sufficient oxygen and nutrient supply, this process depends also on the condition of peribiliary glands in the deeper layers of the bile duct wall, which represent the niche for progenitor cells. Underlining this theory, newly developed biliary epithelial cells can migrate from the peribiliary glands through small connecting canals to the bile duct lumen, where they restore the epithelial lining.

**Figure 2:**
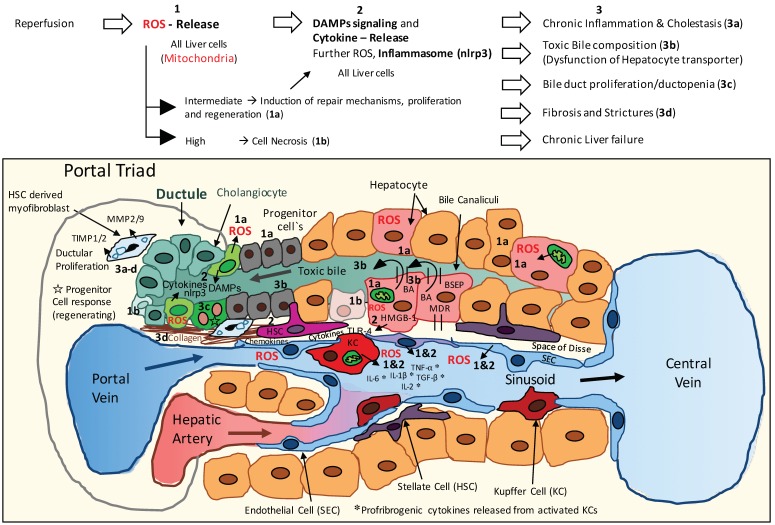
The mechanism of cholangiocyte injury during and after reperfusion in the setting of liver transplantation. The multifactorial mechanisms of injury are described in this figure. Two main drivers are responsible for the development of severe biliary complications: First, at reperfusion, oxygen free radicals are released from the complex of one of the mitochondrial chains in any affected cells, including hepatocytes and cholangiocytes (1). Secondly, the initial oxidative hit triggers downstream inflammation (2) and further aggravation with an ultimate chronic inflammatory status (3). Depending on the liver quality and the amount of graft injury in the donor and during preservation, ATP-dependent bile acid transporters have already an impaired function, which leads to higher vulnerability against toxic bile salts. Such a combination of injuries will likewise lead to an impaired ability to facilitate regeneration of hepatocytes and cholangiocytes (3). BA, Biliary acid; BSEP, Bile salt export pump; MDR, multidrug resistance.

The current main explanation for a higher rate of NAS in DCD transplants is therefore an initial cellular lack of oxygen in hepatocytes and cholangiocytes, leading first to insufficient oxygenation of the progenitor niche with subsequently impaired regeneration of the larger bile ducts [11]. Secondly, during reperfusion, mitochondrial-derived oxidative stresses in biliary epithelial cells trigger the release of reactive oxygen species (ROS) and multiple downstream pathways, including danger signaling, inflammasome activation, and the release of profibrogenic cytokines, which reinforce cholangiocyte and hepatocyte injury (Figure 1 and Figure 2). The third cause of biliary injury are immune-mediated pathways, which depend on blood-type antigens expressed on the biliary epithelia of donor bile ducts, and lead to an ABO antibody-mediated direct injury of biliary epithelial cells (Figure 1). Accordingly, ABO-incompatible liver transplantation has been shown to result in higher rates of NAS [12]. In addition, C-C-Motiv-Chemokin-Receptor 5 Δ 32 (CCR5-Δ 32) -mutation leads to a higher incidence of NAS, due to an impaired attraction of regulatory T cells and dysfunction of the immune defense in the neighborhood of bile ducts [13].

Finally, a disturbed bile salt-phospholipid ratio of bile fluid further destroys sensitive biliary epithelial cells and is caused by insufficient bile salt transporters in hepatocytes, such as Bile salt export pump (BSEP) and multidrug resistance-3 (MDR 3) [14]. ATP-depletion during warm and cold ischemia mainly contributes to this alteration in transporter function. An additionally impaired micelle formation leads to a further decrease of phospholipids in bile, with a subsequent direct bile-salt mediated injury of cholangiocytes, and an increased rate of NAS [11]. Similarly, a decent HCO3- (bicarb) concentration in bile is required to protect cholangiocytes from direct penetration by bile salts and is frequently altered during initial reperfusion [15]. Injured cholangiocytes may also lose their ability to reabsorb a sufficient amount of bile acids. Such features contribute to an increased accumulation of toxic bile salts in cholangiocytes with subsequent cholangiocyte and hepatocyte injury, mainly in larger bile ducts (Figure 1 and Figure 2).

## 3. Risk Factors for Development of Biliary Complications after Liver Transplantation

Before the era of cold storage, ex-situ perfusion of organs had already received particular interest with the aim to maintain organ function outside of the human body by continuously supplying oxygen and nutrients. Yet, with the advent of modern preservation solutions, simple cooling became very attractive and efficient for keeping an organ transplantable for several hours, without severe loss of viability. Accordingly, cold storage remains also nowadays an easy and very successful preservation technique for normal or ideal liver grafts, actually confirmed by a recent benchmark study of cold stored “ideal” primary liver transplants [16]. However, for non-ideal, or so-called marginal liver grafts, the limits of static preservations and techniques have been widely recognized, and machine perfusion techniques have recently been acknowledged for their potential advantages in optimizing organ functions in these grafts. It is notable that definitions of such extended criteria donor livers (ECD) are somewhat arbitrary and include, for example, an advanced donor age of 60–80 years, hepatic steatosis of 15–30 %, and prolonged cold storage of more than 10–12 h [16,17,18,19,20,21]. Importantly, most European centers routinely face liver offers from donors above 60 years of age, together with a significant amount of steatosis in the era of non-alcoholic steatohepatitis (NASH) [22,23], implicating that the “normal” liver graft today is often already aged between 60 and 70 years, with significant macrosteatosis up to 15%, and cold ischemia up to 10 h. Of note, such data differs from the US data, underlined by a significantly lower donor risk index (DRI) [24].

The extended criteria in Europe includes currently liver grafts with high amounts of macrosteatosis (>30 or >40 % (EASL guidelines), prolonged cold ischemia (>12 h), additional donor warm ischemia (DCD), or a very high donor age (>80 years) (EASL guidelines) [25]. Those liver grafts will likewise need optimization before implantation, especially when combined with risky recipients (including retransplantation and high model of end-stage liver disease—MELD score patients) [26].

## 4. General Strategies of Biliary Tree Protection

The general concepts used to improve outcomes in liver transplantations mainly include technical factors, e.g., quick retrievals with short hepatectomy times (below 40 min) [27,28] and extensive bile duct flushes in-situ and ex-situ [27] to remove toxic bile acids [29]. Since well-known risk factors, including donor age [30], donor Body-Mass-Index (BMI) and amount of steatosis [30], and the duration of donor warm ischemia (asystolic warm ischemia < 10 min) [31] are unchangeable, careful selection is the only remaining option to reduce the overall risk. Several transplant programs tend therefore to limit, for example, their cold ischemia to 6 h for DCD and fatty livers [3], and aim to reduce the period between portal vein and arterial anastomosis to supply enough oxygen to the biliary tree as quickly as possible after normothermic reperfusion. It is unclear whether simultaneous portal and arterial reperfusion provides real benefit in terms of biliary injury. In addition, a reduced portal clamping time, by application of a portocaval shunt may protect from bacterial translocation and subsequent LPS-induced aggravation of reperfusion injury [31,32].

## 5. Machine Perfusion

In addition to a reduction of cold and warm ischemia times, ex-situ graft treatment by dynamic preservation techniques appears as a further option to achieve repair of pre-injured liver grafts before implantation. For the livers, two main perfusion approaches are tested in the clinic; either perfusion with blood at physiologic, normothermic or sub-normothermic conditions or perfusion with cooled oxygenated artificial fluids. Normothermic machine perfusion (NMP) aims to replace the cold storage and is applied either in-situ, in donors before procurement (normothermic regional perfusion—NRP), or ex-situ during organ transport [33,34,35,36]. Consecutively, the first randomized controlled NMP trial on livers predominantly donated after brain death (DBD) showed an excellent 1-year patient- and graft- survival rate of 97% [37]. The rates of biliary complications were however not significantly different between the normothermic and cold storage groups and appeared high in both study arms for DCD livers (NAS: 11 vs. 26%, AS: 48 vs. 58%) (Table 1 and Table 2) [37]. Despite the advantages to solving logistical issues, the effects of normothermic machine perfusion (and more specifically normothermic perfusion after cold storage) became less obvious when the cold storage duration of the matched control group was further reduced [38].

Upfront normothermic perfusion already in the donor (NRP) is currently performed in Maastricht III DCD donors in Spain and in the UK with low rates of cholangiopathies [47,48]. One limitation of this approach is the fact that grafts which perform poorly during NRP are discarded, which amounts currently to a rate of approximately 40% non-usable grafts by NRP techniques [56].

**Figure 3:**
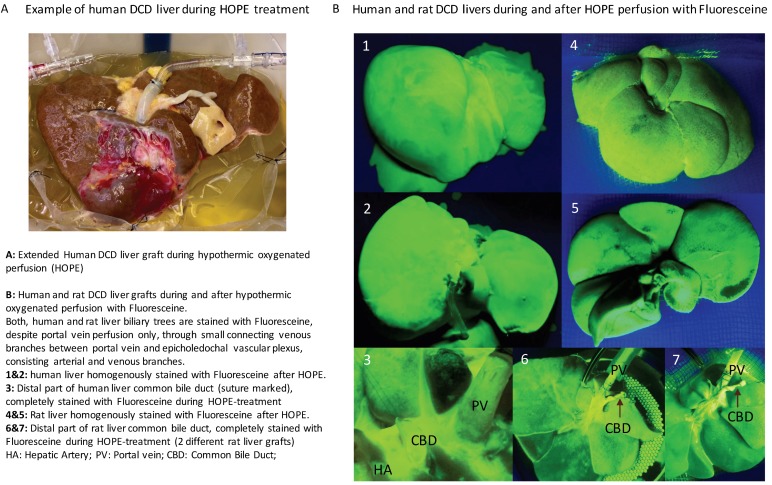
Hypothermic oxygenated perfusion (HOPE) in human and rat DCD livers. (**A**) An example of hypothermic oxygenated perfusion (HOPE) of an extended DCD liver graft prior to transplantation. (**B**) To confirm a complete perfusion with highly-oxygenated perfusate in the cold during HOPE, Fluorescein was added to the perfusate. Both human and rat livers showed a rapid complete perfusion in the cold, despite a low perfusion pressure of 3 mmHg or less. Notably, the entire biliary tree, including the distal tip where the anastomosis is performed with the recipient bile duct, appears also completely stained, which confirms that the perfusate and more importantly the oxygen has reached all liver cells including cholangiocytes. The approval numbers were KEK ZH 2012-1 (January 2012) and KEK ZH 2017-00309 (July 2017), both approved by the Cantonal Ethic Commission in Zurich.

In contrast to an upfront (NRP) or continuous normothermic machine perfusion (NMP) during the entire preservation period, hypothermic oxygenated perfusion (HOPE) is applied end-ischemically after cold storage [26,45,54]. Recent observational studies reported 5-year tumor death-censored patient survival above 95% in extended Maastricht III DCD livers (donated after cardiac death) treated by HOPE [50], comparable to DBD livers. Of note, endischemic HOPE resulted in no graft loss by ischemic cholangiopathy, despite application in high-risk grafts and despite a utilization rate of 90% (Table 1 and Table 2, Figure 3) [50]. HOPE treatment has also been applied recently in Maastricht Type II DCD livers, following initial NRP [51]. Such clinical results in humans were paralleled by experimental studies in livers [43,46] and also in kidneys [57].

Importantly, while the original HOPE treatment is applied only through the portal vein, the group from Groningen currently favors dual hypothermic oxygenated perfusion through the portal vein and the hepatic artery (D-HOPE) [52], and reported the first 20 extended DCD liver grafts with no graft loss in the D-HOPE group compared to un-perfused controls (Table 2) [53]. Randomized trials have been initiated to further evaluate the effect of HOPE and D-HOPE in DBD and DCD liver grafts (Hope-liver.com—Zurich, Groningen Institute for Organ transplantation (GIOT)). Another perfusion approach currently being explored is subnormothermic perfusion, either instead of cold storage or endischemically, and experimental studies have shown promising results in terms of reduction of reperfusion injury and later liver and bile duct histology with subsequently less biliary complications in preclinical studies [39,40,41,42].

Notably, randomized trials comparing concurrent dynamic preservation techniques (NRP vs. normothermic vs. hypothermic) have yet not been performed in any kind of solid organs, and the exact mechanism of protection for each technique remains controversial.

## 6. Suggested Decisive Mechanisms of Machine Liver Perfusion Techniques Against Biliary Injury

Recent research points to three targets for dynamic preservation technology in order to achieve fewer biliary complications after liver transplantation. First, accumulated citric acid metabolites, e.g., mainly succinate, during ischemia have been shown to trigger mitochondrial dysfunction in various tissues including livers, lungs, kidney, brain, and heart [58,59]. Active breakdown of these metabolites before reperfusion would therefore be of major importance to guarantee a well-functioning mitochondrial electron transport during early normothermic reperfusion. A currently unique approach to achieve this task is the introduction of oxygen to the ischemic tissue at cold instead of warm temperature, which results in metabolization of succinate without a concomitant release of reactive oxygen species [60]. Mitochondria are therefore primed by a relatively short phase of hypothermic oxygenated perfusion (HOPE) to function better upon reperfusion under normothermic conditions (Figure 3) [60]. Secondly, cellular energy stores are depleted during any kind of ischemia and need to be uploaded before implantation. Among the major ATP-consuming processes are bile salt transporters, ion channel pumps, and cellular mitotic activities [43]. The regenerative capacity of tissues, including the peribiliary glands, depends therefore on the presence of sufficient levels of phosphorylated nucleotides. Normothermic oxygenated perfusion has been shown to restore liver ATP levels as compared to cold storage (Figure 2) [44,61]. Remarkably though, hypothermic oxygenated perfusion achieves an even higher rate of energy tissue recharging due to a higher ATP yield per oxygen molecule below the Arrhenius breakpoint temperature of 15 °C [61,62,63].

Third, the consequences of normal-functioning mitochondria and uploaded ATP and ADP levels in the first minutes of ischemia reperfusion are multifactorial. These include the prevention of mitochondrial ROS release from complex I during implantation in all liver cells, including cholangiocytes and hepatocytes, and prevention of down-stream inflammasome activation during implantation (Figure 2) [60,64]. It remains unclear whether additional perfusion effects are equally important, such as improvement of arterial microvascular flow by perfusion approaches [46], nutritional support of cholangiocytes [35], and removal of accumulated toxic substances [11,55].

## 7. Prediction of Biliary Complications in Liver Transplantation

A low bile pH (<7.4) during normothermic machine perfusion before implantation serves currently as the best predictor for later ischemic biliary complications (Table 2) [49]. This parallels earlier results from the group of Robert Porte, who suggested to measure bicarbonate in the bile as a surrogate marker of cholangiocyte function [65]. In contrast, lactate clearance, liver enzyme release, and bile production during normothermic machine perfusion were only weak predictors for both graft function and biliary complications [49,66]. Bile pH analysis appears also not reliable during hypothermic perfusion due to insufficient bile production in the cold. Quantification of microRNA from cold flushes or perfusates has been somehow predictive for later biliary complications, but needs further validation [67]. Metabolomic and glyconomics assessments of the cold flush solution and liver biopsies before implantation have been published, but focus solely on the occurrence of primary non-function or early allograft dysfunction [68,69].

## 8. Conclusions and Future Perspective

Machine perfusion technology has a great potential to modify multiple cellular metabolic reactions prior to liver implantation, with significant impact on inflammation and also immune response pathways [60]. In terms of the suggested mechanistic view above, it would be of major importance to determine mitochondrial viability in cells during any machine perfusion approach, instead of analysis of surrogate markers in perfusate or bile. This would clarify whether the perfused liver has already been fully recharged and safely reconditioned to prevent major DAMP signaling and down-stream inflammasome activation during implantation [61,64]. We anticipate, therefore, that in the next few years a better understanding should be available, enabling a perfusion strategy which should be applied in accordance with the type and injury of each liver in order to prevent biliary injury.

## Figures and Tables

**Table 1:** Experimental studies analyzing the impact of machine perfusion on the biliary tree between 2013–2018.

Author	Year	Model	Species	Temp (°C)	Perfusion Duration (h)	Perfusion Route	OLT	Endpoints	Outcome
Goldaracena et al. [39]	2016	DBD	Pig	33, 37	4	PV + HA	Yes	Ischemia-Reperfusion Injury, bile duct injury, liver function (3 day follow up)	Addition of anti-inflammatory substances during sub-and normothermic perfusion improve all endpoints and may reduce biliary injury
Spetzler et al. [40]	2016	DBD	Pig	33	4	PV + HA	Yes	Ischemia-Reperfusion Injury, bile duct injury, liver function, histology	Subnormothermic perfusion improves outcomes after transplantation
Fontes et al. [41]	2015		Pig	21		PV + HA	Yes	Liver function and injury, markers for biliary injury, inflammation and animal survival	Subnormothermic perfusion improves liver function, bile procudetion and survival and reduces the inflammation after liver transplantation, documentation of mediator release (regenerative pathways and inflammation) during subnormothermic perfusion
Knaak et al. [42]	2014	DCD	Pig	33	3	PV + HA	Yes	Endothelial and biliary injury and liver function, animal survival	Endischemic subnormothermic perfusion reduces biliary and endothelial cell injury after transplantation
Schlegel et al. [43]	2013	DCD	Rat	4	1	PV	Yes	Ischemia-Reperfusion Injury, graft function, animal survival, biliary parameters and histology 4 weeks after OLT	HOPE treated DCD livers showed significantly less biliary cirrhosis and fibrosis within 4 weeks after liver transplantation. Such reduced injury is mediated through less reperfusion injury after HOPE treatment
Banan et al. [44]	2016	DBD	Pig	38, gradual rewarming + 38	4–8	PV + HA	No	Markers of hepatocyte injury and biliary tree injury	Reduced biliary epithelial cell injury in gradually rewarmed grafts (rewarming + normotherm better preservation of biliary tree compared to direct normotherm perfusion)
Op den Dries et al. [35]	2016	DBD + DCD	Rat	37	3	PV + HA	No	Markers of biliary function and injury, histology	Normothermic perfusion protects bile ducts when performed instead of cold storage
Westerkamp et al. [45]	2015	DCD	Rat	10, 20, COR	2	PV + HA	No	Markers of biliary function and injury, histology	Less injury of large bile duct epithelium compared to cold storage alone
Liu et al. [36]	2014	DCD	Pig	38	10	PV + HA	No	Markers of biliary function and injury, histology	Normothermic perfusion instead of cold storage improves regeneration of biliary epithelial cells
Op den Dries et al. [46]	2014	DBD + DCD	Pig	10	4	PV + HA	No	Markers of biliary function and injury, histology	Hypothermic perfusion prevents ateriolonecrosis of the peribiliary vascular plexus of the bile ducts

HOPE: Hypothermic oxygenated perfusion; DBD: Donation after brain death; DCD: Donation after circulatory death; PV: Portal vein; HA: Hepatic artery; COR: Controlled oxygenated rewarming, OLT: Orthotopic Liver Transplantation.

**Table 2:** Impact of machine perfusion on biliary complications after liver transplantation (clinical studies, 2015–2018).

Author	Year	Model	n	Technique	Temp (°C)	Device	Perfusion Duration (h)	Perfusion Route	Endpoints	Outcome
Watson et al. [47]	2018	DCD	44	NRP	37	Maquet/ECOPS	2	NRP	Peak ALT, graft function, biliary complications, 90-day survival	NRP is a successful selection tool, DCD livers recovered with NRP showed significant less biliary complications (AS and NAS), no differences in graft survival
Hessheimer et al. [48]	2018	DCD	97	NRP	37	ECMO	2	NRP	graft function, biliary complications, 1-year graft survival	NRP is a successful selection tool, DCD livers recovered with NRP showed significant less biliary complications (AS and NAS), no significant differences in graft survival
Nasralla et al. ✫ [37]	2018	DBD + DCD	121 (34 DCD)	NMP	37	Organox metra	9.1	PV + HA	AST release and 1-year survival after liver transplantation	No difference in biliary complications (AS, NAS), reduced AST release after reperfusion
Watson et al. [49]	2018	DBD + DCD	22 (16 DCD)	NMP	37	Liver Assist	4–6	PV + HA	Post-Reperfusion syndrome, graft function, rate of PNF, biliary complication, bile duct histology and graft survival	25% of transplanted DCD livers developed a NAS, Bile pH during NMP is currently the best predictor for biliary complications at the cutoff 7.5
Bral et al. ✢ [38]	2017	DBD + DCD	9 (4 DCD)	NMP	37	Organox Metra	11.5	PV + HA	Graft function and injury, biliary complications, graft survival	Longer ITU and hospital stay in NMP group
Schlegel et al. [50]	2018	DCD	50	HOPE	10	Liver Assist	2	PV	Post-Reperfusion syndrome, graft function, rate of PNF, HAT and ischemic cholangiopathy, 5-year graft survival	HOPE treated extended DCD liver grafts showed significant improved 5-year graft survival due to less PNF, HAT and ischemic cholangiopathy
De Carlis et al. [51]	2018	DCD (II, III)	15	ECMO + HOPE	37, 10	ECMO/Liver Assist	2, 2	ECMO, PV + HA	Liver function, biliary complications, 1-year survival	No significant differences in biliary complications compared to DBD matching group, 2 NAS (endoscopically treated), no significant differences in survival
Van Rijn et al. [52]	2018	DCD	20	DHOPE	10	Liver Assist	2	PV + HA	Markers of biliary injury including histology of bile ducts	D-HOPE treatment restored hepatic ATP and protects the biliary tree from reperfusion injury and complications
Van Rijn et al. [53]	2017	DCD	10	DHOPE	10	Liver Assist	2	PV + HA	Liver function, ATP content, biliary complications, graft- and patient survival	D-HOPE treatment protect from reperfusion injury and improved 6 and 12 month graft survival and reduced biliary complications
Dutkowski et al. [54]	2015	DCD	25	HOPE	10	Liver Assist	1–2	PV	Graft function, EAD, biliary complications, graft and patient survival	HOPE treated extended DCD liver grafts showed comparable outcomes to matched low-risk primary DBD transplants, biliary complications were reduced compared to untreated DCD liver transplants
Guarrera et al. [55]	2015	ECD (no DCD)	20	HMP	4-8	Medtronic	4–7	PV + HA	Incidence of PNF, EAD, vascular and biliary complication, 1-year graft and patient survival	HMP showed significantly less biliary complications

All studies above are transplant studies, reporting the impact of the perfusion approach on biliary complications as primary or secondary endpoints; n represents the perfused livers or donors, where studies with at least 9–10 perfusions were included; HMP: Hypothermic machine perfusion; HOPE: Hypothermic oxygenated perfusion; DHOPE: Dual-HOPE; ECD: Extended criteria donors; DBD: Donation after brain death; DCD: Donation after circulatory death; PV: Portal vein; HA: Hepatic artery; EAD: Early allograft dysfunction; PNF: Primary non function; HAT: Hepatic artery thrombosis; Perfusion device: J. Guarrera, applied his HMP through a non-pulsatile pump (Medtronic, Minneapolis, MN, USA); AS: Anastomotic strictures; NAS: Non-anastomotic strictures; DCD (II, III): Maastricht II and III category DCD (Italy), 20 min stand off period in donor; NRP: Normothermic regional perfusion in donor; NMP: Normothermic Machine perfusion; AST: Aspartate-Aminotransferase; ✫: Randomized controlled trial, powered for AST release as primary endpoint; Reference [35] includes the first 20 livers of this randomized controlled trial; ✢: NMP livers were matched to cold storage livers, where the control group was of low risk with short cold ischemia time; ECMO: Extracorporeal membrane oxygenation; ALT: Alanine-Aminotransferase .

## Abbreviations

ABO	A and B represent cellular antigens, which initiate production of antithetical circulating antibodies in plasma
ADP	Adenosine diphosphate
ATP	Adenosine triphosphate
AS	Anastomotic strictures
AST	Aspartate-Aminotransferase
CCR5-Δ 32	C-C-Motiv-Chemokin-Receptor 5 Δ 32
DAMP’s	Danger associated molecular pattern’s
DBD	Donation after brain death
DCD	Donation after circulatory death
D-HOPE	Dual Hypothermic oxygenated perfusion
DWIT	Donor warm ischemia time
EAD	Early allograft dysfunction
ECD	Extended Criteria Donor
ECMO	Extracorporeal membrane oxygenation
HA	Hepatic artery
HAT	Hepatic artery thrombosis
HMP	Hypothermic machine perfusion
HOPE	Hypothermic oxygenated perfusion
IC	Ischemic cholangiopathy
KC’s	Kupffer cells
MELD	Model of end stage liver disease
MPT pore	Mitochondria permeability transition pore
NAS	Non-anastomotic stenosis
NRP	Normothermic regional perfusion
NMP	Normothermic machine perfusion
PNF	Primary non function
PV	Portal vein
ROS	Reactive oxygen species
SEC	Sinusoidal endothelial cells
TLR-4	Toll-like-receptor-4
